# Polycomb Repressive Complex 2 Regulates Lineage Fidelity during Embryonic Stem Cell Differentiation

**DOI:** 10.1371/journal.pone.0110498

**Published:** 2014-10-21

**Authors:** Seraphim R. Thornton, Vincent L. Butty, Stuart S. Levine, Laurie A. Boyer

**Affiliations:** 1 Department of Biology, Massachusetts Institute of Technology, Cambridge, Massachusetts, United States of America; 2 BioMicro Center, Massachusetts Institute of Technology, Cambridge, Massachusetts, United States of America; Michigan State University, United States of America

## Abstract

Polycomb Repressive Complex 2 (PRC2) catalyzes histone H3 lysine 27 tri-methylation (H3K27me3), an epigenetic modification associated with gene repression. H3K27me3 is enriched at the promoters of a large cohort of developmental genes in embryonic stem cells (ESCs). Loss of H3K27me3 leads to a failure of ESCs to properly differentiate, making it difficult to determine the precise roles of PRC2 during lineage commitment. Moreover, while studies suggest that PRC2 prevents DNA methylation, how these two epigenetic regulators coordinate to regulate lineage programs is poorly understood. Using several PRC2 mutant ESC lines that maintain varying levels of H3K27me3, we found that partial maintenance of H3K27me3 allowed for proper temporal activation of lineage genes during directed differentiation of ESCs to spinal motor neurons (SMNs). In contrast, genes that function to specify other lineages failed to be repressed in these cells, suggesting that PRC2 is also necessary for lineage fidelity. We also found that loss of H3K27me3 leads to a modest gain in DNA methylation at PRC2 target regions in both ESCs and in SMNs. Our study demonstrates a critical role for PRC2 in safeguarding lineage decisions and in protecting genes against inappropriate DNA methylation.

## Background

Regulation of chromatin structure is a key mechanism for controlling gene expression patterns in response to developmental and environmental cues. Polycomb Group (PcG) proteins play crucial roles in epigenetic gene regulation in all metazoans by modifying chromatin structure. PcG proteins function in a variety of biological pathways including lineage commitment in mammals [1]–[3]. Ablation of any core Polycomb Repressive Complex 2 (PRC2) component, including SUZ12, EED, or EZH2 leads to embryonic lethality in mice during gastrulation, a developmental time point when complex gene expression patterns are established in the embryo [4]–[6]. PRC2 catalyzes tri-methylation of histone H3 lysine 27 (H3K27me3), a modification associated with transcriptional repression [7]. In *Drosophila*, mutations in histone H3 that disrupt K27 methylation lead to phenotypes similar to Polycomb mutants, indicating that H3K27me3 is a crucial mediator of PRC2 function [8]. These data suggest that PRC2 is necessary for regulation of cell fate, yet its role in this process is poorly understood.

Embryonic stem cells (ESCs) have the potential to become any type of cell in the adult organism. This property underpins their utility as a model system to study the mechanisms that drive cell differentiation. In ESCs, PRC2 occupies a large cohort of developmental genes to regulate lineage commitment [9]–[12]. At these genes, H3K27me3 is largely enriched at transcription start sites (TSSs) along with H3K4me3, an activating histone mark associated with Trithorax group (TrxG) proteins [13]–[15]. These “bivalent” promoters are thought to poise genes for later activation during lineage commitment. Bivalent genes in ESCs largely resolve to either an active (H3K4me3 only) or repressed (H3K27me3 only) state during differentiation [14], suggesting that H3K27me3 is critical for both gene repression and for the proper activation of developmental programs during lineage commitment. However, we lack a detailed understanding of how disruption of PRC2 activity in ESCs affects lineage commitment because loss of H3K27me3 leads to a global failure of these cells to properly differentiate.

Emerging evidence suggests crosstalk between PRC2 and the DNA methylation machinery is necessary to ensure proper development. For example, H3K27me3 and DNA methylation are largely exclusive at promoters across human tissues [16] and DNA hypomethylation of bivalent promoters in ESCs appears necessary for maintaining developmental plasticity [17]. Recent studies using Me-DIP showed that loss of H3K27me3 in *Eed^null^* ESCs leads to changes in DNA methylation levels; however, the resolution of this assay was not sufficient to test a direct relationship between these two regulatory pathways [18]. Notably, PRC2 target genes tend to be DNA hypomethylated in cancer cells that show high levels of Polycomb components such as *Ezh2* [19], [20]. While these data suggest that at least in some cases Polycomb activity antagonizes DNA methylation [21], we know little about how their activities are coordinated during lineage commitment. Thus, knowledge of how PRC2 regulates lineage commitment will be critical for understanding its roles in development and how faulty regulation leads to diseases such as cancer.

We investigated the role of PRC2 in regulating gene expression patterns during lineage commitment by analyzing several mutant ESC lines that maintain varying levels of H3K27me3. In particular, we found that a previously described *Suz12* gene trap (*Suz12^GT^*) ESC line [22] maintained intermediate levels of H3K27me3 and was able to undergo directed differentiation *in vitro,* albeit less efficiently compared to wild-type cells [23]. This result is in contrast to *Suz12* truncation (*Suz12^Δ^*) or *Eed* point mutant (*Eed^null^*) ESCs that show near-complete loss of the mark and an inability to differentiate [10], [24]. Using this set of genetic tools, we demonstrate that proper H3K27me3 levels are necessary for both activation of lineage programs and for repression of alternate pathways to maintain lineage fidelity during directed differentiation of ESCs toward spinal motor neurons (SMNs). We next analyzed changes in DNA methylation levels in *Suz12^GT^* cells during SMN differentiation at nucleotide resolution and found that loss of H3K27me3 directly led to a modest gain in DNA methylation at PRC2 target regions. While disruption of normal DNA methylation levels did not lead to further changes in expression of PRC2 targets in *Suz12^GT^* cells, we propose that a low-level gain of DNA methylation at promoters may lead to further epigenetic instability. Thus, our findings indicate that PRC2 activity is necessary to maintain cell fate plasticity and lineage fidelity during differentiation, and may safeguard developmental genes against more permanent repression.

## Results

### PRC2 mutant ESC lines maintain varying levels of H3K27me3

**Figure 1:**
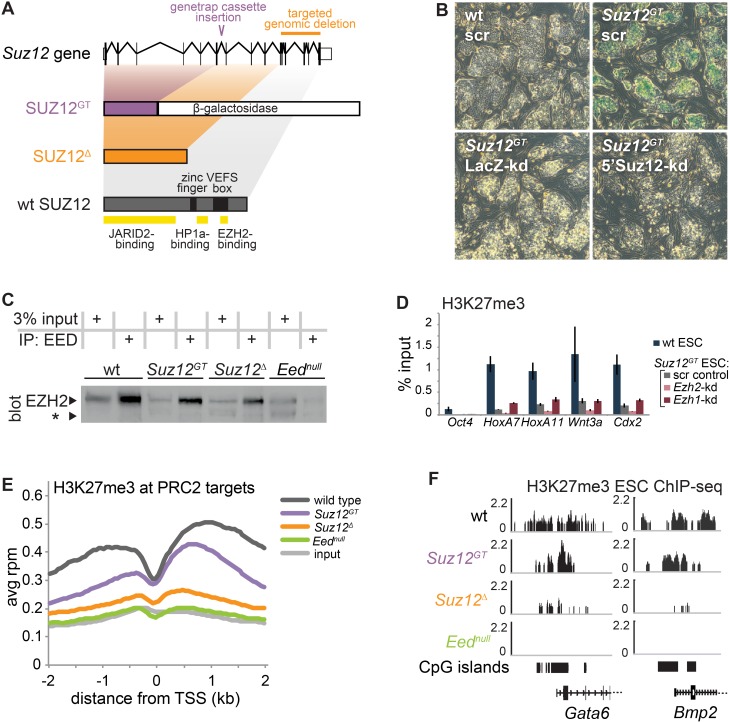
Comparison of PRC2 mutant ESC lines. (**A**) At top, a diagram of the structure of the wild-type (wt) *Suz12* gene. Below, the proteins encoded by the two mutant alleles used here (SUZ12^GT^ and SUZ12*^Δ^*) and the wt allele are shown to scale, and domains of interest are noted on wt SUZ12. (**B**) X-gal staining was performed on wt ESCs (upper left) and *Suz12^GT^* ESCs (upper right) expressing either a scrambled control hairpin, a hairpin targeted to *LacZ* (encoding β-galactosidase) (lower left), or a hairpin targeted to the 5′ end of *Suz12* (lower right). (**C**) Immunoprecipitation of EED was performed in wt, *Suz12^GT^*, *Suz12^Δ^*, and *Eed^null^* ESCs. The samples, including 3% input, were subjected to SDS-PAGE. EZH2 immunoblot was performed as indicated by the labeled band (left). EZH2 degradation product is marked by an asterisk (*). (**D**) ChIP-qPCR for H3K27me3 was performed on wt and *Suz12^GT^* ESCs expressing hairpins: scr (scrambled control), *Ezh2*-kd (targeted to *Ezh2*), and *Ezh1*-kd (targeted to *Ezh1*). All genes tested except *Oct4* are PRC2 target genes. Error bars show standard deviation of three technical replicates. In (**E**) and (**F**), ChIP-seq for H3K27me3 was performed on wt, *Suz12^GT^*, *Suz12^Δ^*, and *Eed^null^* ESCs. ChIP-seq datasets are normalized to the total mapped reads. (**E**) A metagene analysis of H3K27me3 ChIP-seq enrichment is shown across the average of all PRC2 target genes +/− 2 kb relative to the TSS for wt, *Suz12^GT^*, *Suz12^Δ^*, and *Eed^null^* ESCs, as well as input. (**F**) H3K27me3 ChIP-seq tracks in ESCs. Representative examples of PRC2 target promoters (*Gata6* and *Bmp2*) showing H3K27me3 levels in *Suz12^GT^*, *Suz12^Δ^*, and *Eed^null^* ESCs.

PRC2 catalyzes H3K27me3, and its recruitment to a large cohort of developmental genes in ESCs suggests a critical role for PRC2 in regulating lineage commitment [9], [10], [25]. Because PRC2 mutant ESCs are unable to undergo proper directed differentiation, how PRC2 regulates gene expression patterns during lineage commitment is poorly understood. Our recent work found that a *Suz12* genetrap ESC line that creates a SUZ12-β-galactosidase fusion (denoted here as *Suz12^GT^*) (Figure 1A and S1A–B) [22] maintained H3K27me3, albeit at lower levels compared to wild-type cells [23]. Importantly, while these cells exhibit impaired differentiation, H3K27me3 levels in *Suz12^GT^* cells can be rescued by exogenous expression of wild-type *Suz12* indicating that the activity of the complex can be restored to normal levels [26]. By comparison, another *Suz12* mutant ESC line (denoted here as *Suz12^Δ^*) generated by truncation of SUZ12 (Figure 1A and S1A–B) and an ESC line that harbors a point mutation in *Eed* (denoted *Eed^null^*) exhibit near complete loss of bulk H3K27me3 by immunoblot, as well as differentiation defects [10], [27]. Thus, we analyzed the pattern of H3K27me3 enrichment by ChIP-Seq in mutant and wild-type ESCs to determine chromatin enrichment for this mark across the genome. Close inspection of biological replicates showed that while H3K27me3 displayed lower average levels at PRC2 target genes in *Suz12^GT^* ESCs, its pattern of enrichment at TSSs is highly similar to wild-type ESCs (Figure 1E–F and S1G–H). In contrast, H3K27me3 is largely diminished across the genome in both *Suz12^Δ^* and *Eed^null^* ESCs.

Our observation that the *Suz12^GT^* ESCs display detectable H3K27me3 levels suggests that at least a partially active PRC2 complex can function in these cells. To test whether we could recover canonical PRC2 from mutant ESC lines, we first confirmed the expression of the *Suz12*-β-gal fusion in ESCs by X-gal staining (Figure 1B). We next immunoprecipitated PRC2 with an EED-specific antibody and resolved the complexes by SDS-PAGE, followed by immunoblotting for EZH2. We observed that EED interacted with EZH2 in the mutant ESCs, and that EZH2 appeared slightly more stable in *Suz12^GT^* ESCs compared to the *Suz12^Δ^* or *Eed^null^* lines, as shown by the less prominent degradation product (Figure 1C, S1C–E). Prior studies have indicated that EZH1, another H3K27-methyltransferase, can partially rescue loss of EZH2 in ESCs by forming an alternate form of PRC2 [28], [29]. Thus, we also tested the possibility that H3K27me3 levels were maintained in *Suz12^GT^* ESCs through an EZH1-PRC2 complex. Whereas H3K27me3 levels were further diminished in *Suz12^GT^* ESCs upon shRNA-depletion of *Ezh2*, *Ezh1* suppression did not affect H3K27me3 levels at target genes, as measured by ChIP-qPCR (Figure 1D and S1F). Collectively, these data suggest that the *Suz12^GT^* allele functions as a hypomorph *in vitro* and can be used as a tool to study the role of PRC2 in lineage commitment.

### H3K27me3 is necessary for lineage specification

**Figure 2:**
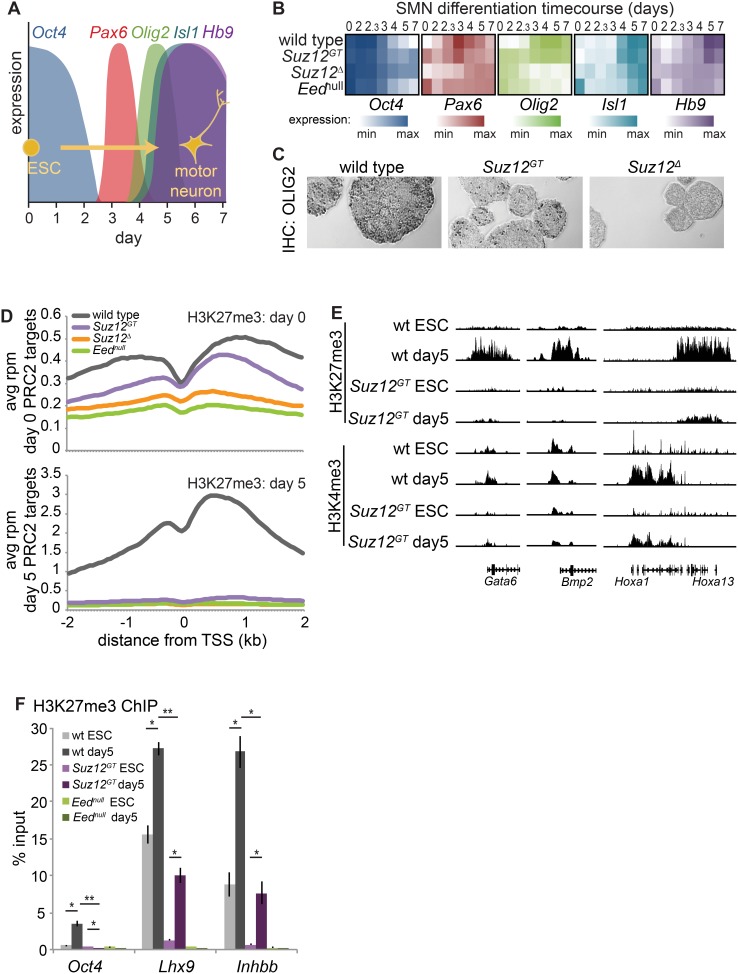
H3K27me3 levels show differences across SMN differentiation in *Suz12GT* cells compared to wt cells. (**A**) Cartoon showing changes in marker expression across the spinal motor neuron (SMN) differentiation time course. (**B**) Heatmap of qRT-PCR analysis of genes from (A). White: minimum expression; saturated color: maximum expression level observed for that gene. Expression for each of the five genes is shown, log_2_ transformed, for wild-type (wt) (top), *Suz12^GT^* (2^nd^), *Suz12^Δ^* (3^rd^), and *Eed^null^* (bottom) cells. The time course progresses from left to right for 7 days. (**C**) IHC for OLIG2 on paraffin-embedded sectioned day 5 SMNs. OLIG2 expression is shown as darkly stained cells. (**D**) ChIP-seq enrichment for H3K27me3 is shown for wt, *Suz12^GT^*, *Suz12^Δ^*, and *Eed^null^* ESCs and corresponding day 5 differentiated cells. ChIP-seq datasets are represented as metagene plots showing average reads per million within 2 kb of all TSSs for wt, *Suz12^GT^, Suz12^Δ^,* and *Eed^null^* cells. Day 0 (ESC) is at top, while day 5 SMN is shown at bottom. (**E**) H3K27me3 and H3K4me3 ChIP-seq tracks for *Gata6* promoter (left); *Bmp2* promoter (middle); and the *HoxA* cluster (right) show that H3K27me3 levels change (increase, decrease, or stay the same, depending upon locus) in *Suz12^GT^* cells upon differentiation. H3K4me3 levels change similarly in *Suz12^GT^* and wild-type cells over differentiation, but at lower levels in *Suz12^GT^*. (**F**) ChIP-qPCR data confirm that *Suz12^GT^* cells are capable of gaining significant H3K27me3 at *Lhx9* and *Inhbb*, the two genes that gain the most H3K27me3 over differentiation in wt cells according to the ChIP-Seq data, whereas *Eed^null^* cells show no gain in H3K27me3 at these genes. Error bars represent standard deviation of three technical replicates. P-values were calculated with a Student’s two-sided *t*-test. *: p<5E-10; **: p<5E-15.

Understanding the role of PRC2 during lineage commitment has been a challenge because ESCs lacking H3K27me3 do not properly differentiate. Thus, we investigated this question using the various PRC2 mutant ESC lines described above. As a model of lineage commitment, we performed directed differentiation of ESCs to Spinal Motor Neurons (SMNs) by removal of LIF as well as addition of retinoic acid and an agonist of the *Sonic Hedgehog* signaling pathway [30] (Figure 2A). We found that genes normally activated in differentiating SMNs (e.g. *Pax6*, *Olig2*, *Isl1*, and *Hb9*) were expressed in a similar temporal manner in *Suz12^GT^* cells albeit at lower levels compared to wild-type cells (Figure 2B). In contrast, these genes failed to properly activate in *Suz12^Δ^* or *Eed^null^* cells. Consistent with these data, immunohistochemistry showed that OLIG2, a PRC2 target and key transcription factor that directs SMN differentiation, was detected in a proportion of *Suz12^GT^* cells at day 5 of differentiation, but not in *Suz12^Δ^* cells (Figure 2C). In agreement with previously reported results, we also observed that the pluripotency marker *Oct4* showed a delayed repression in PRC2 mutant cells during differentiation compared to wild-type cells [22] (Figure S2A). Thus, *Suz12^GT^* ESCs maintain the ability to activate lineage programs, albeit less efficiently than wild-type cells suggesting a critical role for H3K27me3 in this process.

Bivalent genes in ESCs largely resolve to either an active (H3K4me3 only) or repressed (H3K27me3 only) state during differentiation [14]. In addition, PRC2 target genes tend to gain large H3K27me3 domains at non-lineage genes during differentiation [19]. Thus, we next tested how H3K27me3 levels in ESCs can affect chromatin states in SMN differentiation. We observed that about half of PRC2 target genes in ESCs showed higher enrichment of H3K27me3 by day 5 of SMN differentiation in wild-type cells (Figure 2D, E and S2C). Of the 1670 bivalent genes that gained over 4-fold in H3K27me3 levels in wild-type cells during differentiation, 836 (50%) also showed a concomitant decrease greater than 1.5 fold in H3K4me3 levels. This is consistent with the idea that a subset of bivalent genes resolves to a more repressed state (Figure 2E, S2C left). Gene ontology (GO) analysis indicated that genes that gained H3K27me3 function in transcription, neuronal differentiation (e.g. genes of non-SMN lineages), pattern specification, and embryonic morphogenesis, among other biological pathways important for proper lineage specification (Table S4 in File S1). Of the 1226 genes that gained 1.5-fold H3K27me3 over differentiation in wild-type cells and were also bivalent in *Suz12^GT^*, 79 also showed increase in *Suz12^GT^* over differentiation, albeit at 2.56-fold reduced levels. Notably, 63/79 (79.7%) also lost H3K4me3>1.5-fold, resolving their bivalency in favor of H3K27me3. (Figure 2E and S2C right). Additionally, we confirmed that the genes that gained the most H3K27me3 in wild-type cells also showed an increase in *Suz12^GT^* cells, albeit at considerably lower levels (Figure 2D). H3K27me3 enrichment in *Suz12^GT^* SMNs more closely paralleled the levels observed in wild-type ESCs whereas *Suz12^Δ^* or *Eed^null^* mutant cells showed no H3K27me3 as expected (Figure 2D, F, S2B).

Bivalent PRC2 target genes that are needed during lineage specification resolve their bivalency in favor of H3K4me3. Of the 368 bivalent genes that lost over 2-fold in H3K27me3 levels in wild-type cells over differentiation, 138 (37.5%) also showed a concomitant increase greater than 2-fold in H3K4me3 levels (Figure 2E and S2C, left). GO analysis of genes losing H3K27me3 revealed enrichment for genes that have roles in cell adhesion (e.g. *cadherins* and *protocadherins*), neuron differentiation (e.g. *HoxA1*, *HoxA2*, *Sox1*), and axon guidance (e.g. *Gap43*, *Sema6c*), consistent with the progressive activation of the SMN pathway (Table S4 in File S1). Notably, of the 64/368 genes that lost 2-fold H3K27me3 over differentiation in wild-type cells and were also bivalent in *Suz12^GT^*, 49 also showed a decrease over differentiation in *Suz12^GT^*, albeit at 2.3 fold reduced levels, and 36/79 (45.6%) also gained H3K4me3>2-fold, resolving their bivalency in favor of H3K4me3 (Figure S2C, right). GO analysis revealed a broad spectrum of functions for genes losing H3K27me3 over differentiation in *Suz12^GT^* cells (Table S4 in File S1), suggesting that additional pathways failed to be repressed in the mutant cells. Together, these data suggest that in ESCs, PRC2 is critical for establishing chromatin states that allow for proper lineage specification.

### H3K27me3 is necessary for lineage restriction and fidelity

**Figure 3:**
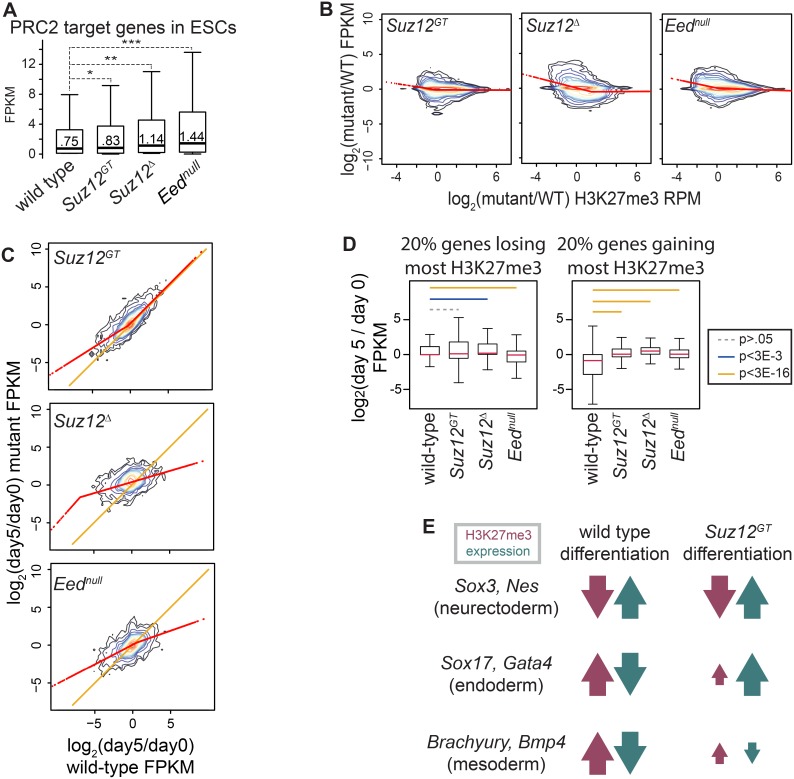
Proper H3K27me3 levels are necessary for coordinating developmental gene expression programs. (**A**) RNA-seq of wild-type (wt), *Suz12^GT^*, *Suz12^Δ^*, and *Eed^null^* ESCs. Distributions of FPKMs of PRC2 target genes are shown as box and whisker plots that extend from the 25^th^ to 75^th^ percentile; whiskers represent 1.5x the length of the box. P-values were calculated with Student’s two-sided *t*-test. *: p<5E-2; **: p<5E-5; ***: p<5E-7. (**B**) RNA-seq and H3K27me3 ChIP-seq are shown for *Suz12^GT^* (left panel), *Suz12^Δ^* (middle panel), and *Eed^null^* (right panel) ESCs with respect to wt. Kernel densities of the data are represented along 14 levels. A segmented regression method was used to calculate localized best-fit and is plotted in red. (**C**) RNA-seq of wt, *Suz12^GT^*, *Suz12^Δ^*, and *Eed^null^* ESCs and day 5 SMNs. y-axis shows log_2_ of the ratio of FPKM in differentiated: ESC in mutant lines as indicated; x-axis represents this ratio in wt cells. Kernel densities of the data are represented along 14 levels. Segmented regression on the data is plotted in red and the y = x line in orange. (**D**) Relationship between change in H3K27me3 and expression over differentiation is shown as box plots. All genes were binned by change in H3K27me3 levels over differentiation in each respective cell type (log_2_ of H3K27me3 (day 5/day 0)). y-axis shows distribution of change in expression (log_2_ of FPKM (day 5/day 0)). **Left panel:** bottom quintile in each cell type. **Right panel:** top quintile in each cell type. P-values were calculated using a Student’s t-test and are represented by colored lines between bins. (**E**) Change in H3K27me3 and expression over differentiation for representative genes. Gain or loss in H3K27me3 or expression is represented by upward or downward arrow, respectively, whereas magnitude is represented by size of arrow.

While studies have shown that PRC2 target genes are de-repressed in PRC2 mutant ESC lines, how changes in H3K27me3 levels impact gene expression during differentiation is largely unknown. Thus, we performed RNA-Seq on *Suz12^GT^*, *Suz12^Δ^*, *Eed^null^*, and wild-type ESCs and compared differences in expression patterns with changes in H3K27me3 levels. As expected, PRC2 target genes are expressed at a higher level in *Eed^null^* (median = 1.44 fpkm; p<5E-7) and *Suz12^Δ^* ESCs (median = 1.14 fpkm; p<5E-5) that lack H3K27me3 compared to wild-type ESCs (median = 0.75 fpkm) (Figure 3A). Expression of genes in *Suz12^GT^* ESCs is more similar to wild-type, albeit slightly higher (median = .83 fpkm; p<5E-2), consistent with the partial maintenance of H3K27me3 in these cells. Differences in overall gene expression between the mutant and wild-type cells were largely due to altered regulation of PRC2 target genes, as demonstrated by the similar changes observed when considering all genes (Figure S3A). Notably, genes that displayed the most significant loss of H3K27me3 in PRC2 mutants relative to wild-type ESCs correlated with the highest increase in expression levels, as shown by the regression line (Figure 3B; see also S3B for alternative regression methods). These data strongly suggest that H3K27me3 levels are proportional to PRC2 target gene repression in ESCs.

We next analyzed how H3K27me3 levels impact gene expression states during directed SMN differentiation. As expected, the expression profiles of *Eed^null^* and *Suz12^Δ^* cells did not show global activation of the SMN gene expression program, consistent with their inability to undergo directed differentiation (Figure 3C, middle and bottom, and Figure S3C). This trend was also observed when examining only PRC2 target genes (Figure S3D). *Suz12^GT^* cells, however, showed overall global activation of lineage-specific genes during differentiation, as shown by the clustering of the data points around the x = y line in the upper right corner of the plot. In contrast, many of the genes down-regulated in wild-type cells failed to be properly repressed in *Suz12^GT^* cells, as indicated by the regression line on the left-hand side of the plot (Figure 3C, top panel and Figure S3C).

To more precisely quantify the relationship between changes in H3K27 tri-methylation levels and expression of PRC2 target genes during SMN differentiation, we binned all genes into quintiles based on fold change in H3K27me3 levels over differentiation, and the change in expression was plotted for the bottom (Figure 3D, left) and top (Figure 3D, right) quintiles for each cell type. In wild-type cells, the set of genes that gained the most H3K27me3 over differentiation displayed the largest change in expression, showing significantly lower expression than the other quintiles of genes (Figure S3E). GO analysis indicated that target genes in this category have roles in transcription regulation, pattern specification, embryonic morphogenesis, neuronal differentiation (e.g. genes of non-SMN neuronal lineages), and cell fate commitment (Table S4 in File S1).

In contrast, the genes that gained some H3K27me3 in *Suz12^GT^* cells failed to show a similar decrease in expression as compared to wild-type cells during differentiation (Figure 3D, right). Specifically, these cells were unable to repress genes expressed in other germ layers such as *Sox17*, *Gata4*, *T*, and *Bmp4* that were normally silenced during lineage commitment in wild-type cells (Figure 3E, S3G). On the other hand, the top 20% of genes that *lose* H3K27me3 over the course of differentiation in wild-type cells showed a similar relative increase in expression in both wild-type and *Suz12^GT^* cells (Figure S3F). This set of genes functions in cell adhesion, regionalization, axon guidance, and neuron differentiation including genes important for SMN differentiation and function (Table S4 in File S1). *Eed^null^* and *Suz12^Δ^* cells showed no strong directional change in expression in either of these quintiles, in agreement with their failure to undergo proper directed differentiation (Figure 3D). Thus, proper H3K27me3 levels are necessary both for activation of lineage programs and for maintenance of lineage fidelity by repressing inappropriate developmental pathways.

### PRC2 activity antagonizes DNA methylation in *cis* during lineage commitment

**Figure 4:**
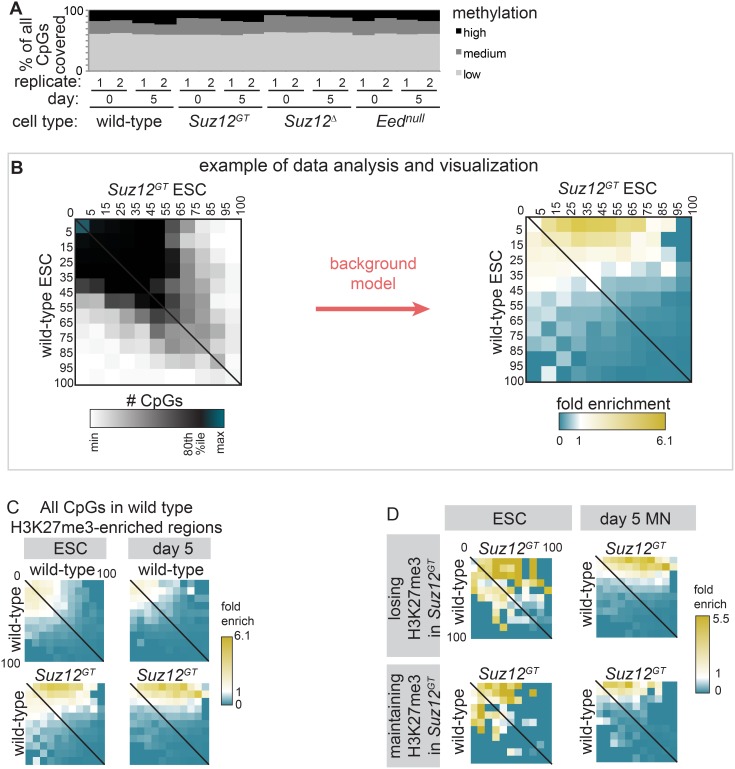
PRC2 is antagonistic to DNA methylation in *cis*. Through RRBS, percent methylation at each CpG with ≥10-fold coverage was calculated in wild-type (wt), *Suz12^GT^*, *Suz12^Δ^*, and *Eed^null^* ESCs and day 5 differentiated cells. (**A**) Distribution of methylation at all CpGs is shown. Low: ≤15% methylated; high: ≥80%. (**B**) This panel is an explanatory example of the data analysis and visualizations used in Figures 4C–D, using the lower-left heatmap of 4C as an example. (**left**) CpGs were binned according to % methylation in wt (y-axis) and *Suz12^GT^* (x-axis) ESCs. Thus, the matrix displays the number of CpGs in each 2-D bin. The data is largely along the x = y line (CpGs with the same % methylation in *Suz12^GT^* as wild-type), shifting towards the top right (more methylation in *Suz12^GT^*). (**right**) Fold enrichment over the overall distribution of the data was determined for each bin using a replicate-based background model (see Methods). In this example, high statistical enrichment over background in *Suz12^GT^* cells (yellow) is visible for CpGs with little methylation in wt cells (**C**) CpGs in wt H3K27me3-enriched regions are used to analyze changes in DNA methylation in ESCs (left panel) and day 5 SMN (right panel). The enrichment in the lower heatmaps shows CpGs with low methylation in wt (y-axis) gaining methylation in *Suz12^GT^* (x-axis). (**D**) (Top) H3K27me3-enriched regions in wt ESCs that lose enrichment in *Suz12^GT^*. (Bottom) Regions maintaining H3K27me3 enrichment in *Suz12^GT^* ESCs. Regions losing H3K27me3 in *Suz12^GT^* cells gain overall more DNA methylation than those maintaining significant H3K27me3.

Emerging evidence indicates that PRC2 functions with other epigenetic modifiers to regulate differentiation. For example, a recent study using meDIP-chip (ChIP for 5-methyl-cytosine coupled with a promoter microarray) suggested that DNA methylation levels were modestly affected in *Eed^null^* ESCs compared to wild-type cells [18], however, this method measures DNA methylation levels over hundreds of base pairs, making it difficult to determine a direct relationship between these complexes. Moreover, how these two pathways are coordinated to regulate gene expression has not been examined during lineage commitment. Thus, we performed reduced representation bisulfite sequencing (RRBS) [31], [32] during SMN differentiation as a method to analyze DNA methylation at individual CpG sites across the genome in wild-type and PRC2 mutant cells. While cell lines lacking all H3K27me3 were slightly hypomethylated in general, we did not observe dramatic changes in global DNA methylation over differentiation in wild-type cells or in any of the PRC2 mutant cells (Figure 4A). However, by focusing on individual CpG sites within PRC2 target regions, we detected modest but significant changes in DNA methylation.

To further analyze the relationship between H3K27me3 and DNA methylation, we limited our analysis to those CpGs with ≥10x coverage and within H3K27me3-enriched regions defined in wild-type cells in either ESC or day 5 SMNs. Figure 4B is a diagram of the data analysis and visualization methods we used for this comparison. In this example, first, each CpG was binned according to its percent DNA methylation in wild-type ESCs on the y-axis, and according to its percent DNA methylation in *Suz12^GT^* ESCs on the x-axis (Figure 4B, left). The results can be displayed as a heatmap showing the number of CpGs in each 2-D bin. Fold enrichment relative to a background model was then determined for each bin (see Methods) (Figure 4B, right). Overall, we found that CpG sites within H3K27me3-enriched regions showed very low levels of DNA methylation. However, in ESCs and at day 5 of SMN differentiation, PRC2 mutants displayed a significantly larger-than-expected number of CpGs with increased levels of DNA methylation compared to wild-type cells (p<5E-7), as shown by the signal at the top-right corner of the bottom two heatmaps in Figure 4C (depicting *Suz12^GT^*) and Figure S4. We also found that CpG sites that are within regions that lose H3K27me3 in *Suz12^GT^* ESCs or day 5 SMNs gained more DNA methylation compared to regions that maintained H3K27me3 in these cells (Figure 4D). These results indicate that PRC2 (or H3K27me3) directly antagonizes DNA methylation, and loss of the mark allows increased DNA methylation.

### Modest increase in DNA methylation in PRC2 mutants does not affect gene expression

We next wanted to test the consequence of the increase in DNA methylation on expression of PRC2 target genes in *Suz12^GT^* cells. To address this question, CpG dinucleotides in regions losing H3K27me3 enrichment in *Suz12^GT^* cells were first assigned to a gene based on distance and position relative to the nearest transcription start site (see Methods). We then determined the change in expression at those genes compared to wild-type cells using our RNA-seq data. In ESCs, about 25% of CpG sites in H3K27me3-depleted regions gained ≥10% methylation in *Suz12^GT^* ESCs, and mapped to genes including *Bmp2*, *Gata3*, and *Fgf8*, as well as a number of homeobox genes. In day 5 SMNs, about 18% of CpG sites in H3K27me3-depleted regions gained ≥10% DNA methylation in *Suz12^GT^* cells, and are associated with genes such as *En1* and *Wnt6*. We also observed that a smaller proportion of CpG sites displayed a ≥10% decrease in DNA methylation within these regions. While we observed a trend toward an overall increase in DNA methylation at CpG sites within PRC2 target regions, individual CpGs in proximity to a given gene can either gain or lose DNA methylation in *Suz12^GT^* cells suggesting that changes in DNA methylation are stochastic.

**Figure 5:**
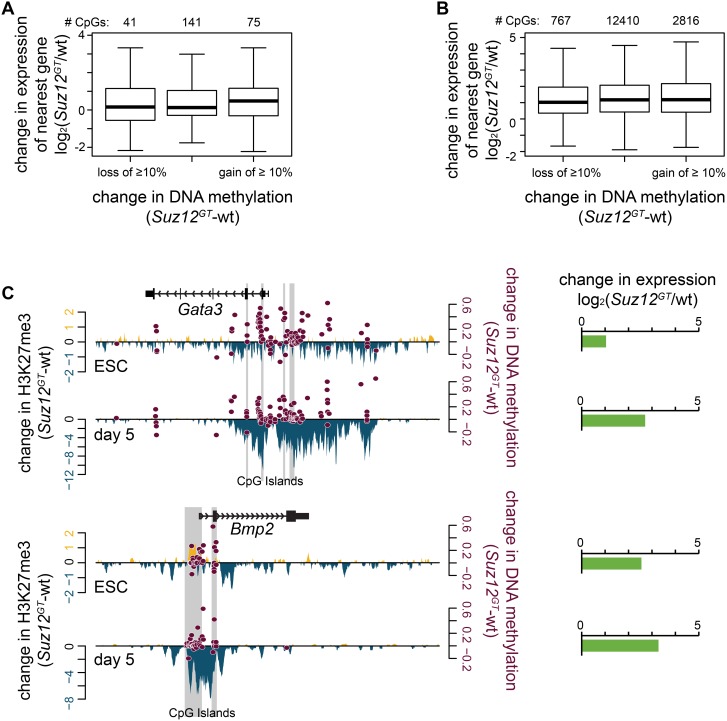
Increased DNA methylation upon loss of PRC2 does not lead to target gene repression. (**A**) Regions with significantly enriched H3K27me3 in wild-type (wt) ESCs were considered. All CpGs in these regions with 10x coverage via RRBS in both wt and *Suz12^GT^* ESCs, and ≥.1 FPKM in at least one of these cell types, were used in the analysis. Of these 257 CpGs, 41 lose ≥10% DNA methylation, 75 gain ≥10% DNA methylation, and 141 do not change. The distribution of change in expression in *Suz12^GT^* ESCs with respect to wt ESCs of the genes associated with these CpGs is plotted on the y-axis. No association between change in DNA methylation and gene expression is observed. (**B**) Same as in (A) except in day 5 SMNs. Of these 15993 CpGs, 767 lose ≥10% DNA methylation, 2816 gain ≥10% DNA methylation, and 12410 do not change. The distribution of change in expression in *Suz12^GT^* cells with respect to wt cells of the genes associated with these CpGs is plotted on the y-axis. No association between change in DNA methylation and gene expression is observed. (**C**) Two example genes, *Gata3* and *Bmp2*, are shown here. Change in H3K27me3 signal between wt and *Suz12^GT^* cells is plotted on the left y-axis; gain in *Suz12^GT^* is shown in yellow, and loss is shown in blue. Change in DNA methylation for each CpG with 10x coverage in both cell types is plotted in maroon on the right y-axis. Change in gene expression (log_2_ of the ratio of the FPKMs (*Suz12^GT^*/wt)) is shown in horizontal bar graphs to the right of the panel.

By comparison of RNA-Seq data sets in wild-type and *Suz12^GT^* ESCs or day 5 differentiated cells, we did not observe a significant change in expression for genes that showed either an overall modest increase or decrease in DNA methylation at these sites (Figure 5A–B). Figure 5C shows representative examples of PRC2 target genes and their observed changes in H3K27me3, DNA methylation, and gene expression in *Suz12^GT^* cells. For example, we observed overall loss of H3K27me3, gain of DNA methylation, and increased expression of *Gata3* and *Bmp2* in *Suz12^GT^* ESCs and over the differentiation time course compared to wild-type cells. Notably, the observed derepression at PRC2 target genes in *Suz12^GT^* cells is similar in magnitude to genes that do not display changes in DNA methylation, suggesting that the modest increase in DNA methylation does not suppress the effects of loss of PRC2 activity. Collectively, our data suggest that PRC2 plays a role in preventing inappropriate DNA methylation at lineage-specific genes and that developmental promoters are largely regulated by PRC2 activity during lineage commitment.

## Discussion

Regulation of PRC2 activity is essential to mammalian development and differentiation. Loss of PRC2 and its catalyzed mark, H3K27me3, leads to lethality during gastrulation, a period of development when complex gene expression patterns are established in the embryo [4], [6], [27]. While studies have shown that PRC2 silences developmental programs in ESCs, its roles during differentiation and lineage commitment have not been extensively studied due to the inability of PRC2 mutant ESCs to properly differentiate. In order to gain new insights, we exploited a mutant genetrap allele of *Suz12* that acts as a hypomorph *in vitro* in that it maintains partial H3K27me3 levels and allows for low-efficiency directed differentiation of ESCs to SMNs. We show that the proper lineage programs can be activated during differentiation, however, genes that normally gain H3K27me3 over differentiation and become repressed in wild-type cells failed to be fully repressed in *Suz12^GT^* cells. Comparatively, *Eed^null^* and *Suz12^Δ^* ESCs lack H3K27me3 and fail to properly induce differentiation programs. These data provide strong evidence that regulation of proper H3K27me3 levels is necessary for lineage commitment.

H3K4me3 and H3K27me3 co-occur at the promoters of a large cohort of developmental regulators in ESCs [14]. These “bivalent” promoters are thought to poise genes for later activation during lineage commitment. While bivalent domains largely resolve to either active (H3K4me3 only) or repressed (H3K27me3 only) during lineage commitment, current evidence suggests that together the two histone marks are necessary to coordinate temporal activation of lineage programs when signaled to do so. For example, PRC2 is thought to be critical for repression by inhibiting elongation or by preventing RNA Pol2 binding in mammalian cells [33]. Upon activation, genes maintain H3K4me3 and gain H3K36me3 marks which are poor substrates for PRC2 [34]–[36]. Consistent with these observations, loss of PRC2 (and H3K27me3) in ESCs has been shown to lead to de-repression of target genes as well as a failure to activate lineage pathways [9], [10]. The correlation between a gain in H3K27me3 and the repression of non-lineage pathways supports a functional role for H3K27me3 in mediating lineage fidelity. Consistent with this idea, we find that while lower H3K27me3 levels in *Suz12^GT^* ESCs allow for proper temporal activation of lineage genes, these cells failed to accumulate larger H3K27me3 domains sufficient to repress non-lineage genes. Together, these data indicate that PRC2 is necessary for both the proper induction of lineage programs and for repression of alternate pathways to restrict cell fate.

CpG dinucleotides at the promoters of developmental genes, which often reside in CpG islands, are primarily unmethylated in the genome. While transcription factor binding appears to be a major mechanism for preventing DNA methylation, our data also support a role for PRC2 in antagonizing DNA methylation during lineage commitment. Consistent with this idea, accumulating evidence indicates that repression of developmental genes is largely regulated by H3K27 methylation and not DNA methylation [37]. Our findings that *Suz12^GT^* ESCs can differentiate, albeit less efficiently, and that these cells harbor regions of variable H3K27me3 levels compared to wild-type cells, make them an important tool to investigate this relationship. Using RRBS, we show that in *Suz12^GT^* ESCs, PRC2 targets losing H3K27me3 with respect to wild-type cells were more likely to gain DNA methylation at CpG sites in these regions compared to regions that maintained H3K27me3 levels, providing the first direct evidence that PRC2 activity is directly antagonistic to DNA methylation in *cis*.

What targets a gene for permanent repression or activation? PRC2 has recently been shown to recruit TET1, a dioxygenase that converts 5-methyl-cytosine into 5-hydroxymethyl-cytosine, which may safeguard developmental genes against inappropriate DNA methylation [38]. *Tet1* knockout animals display epigenetic abnormalities, but its loss does not impact embryonic or postnatal survival [39], suggesting that other family members (e.g. TET2 and TET3) or mechanisms also contribute to regulating DNA methylation levels at PRC2 target genes. Additionally, a recent study showed that PRC2 recruits DNMT3L, a catalytically inactive DNA methyltransferase that sterically competes with active DNA methyltransferases to prevent DNA methylation at PRC2 target sites [17]. It is possible that loss of PRC2 activity in *Suz12^GT^* cells prevents localization of TET family members or DNMT3L to promoters, leading to inappropriate DNA methylation. Each of these mechanisms could be critical for safe-guarding developmental genes from an increase in DNA methylation that could ultimately lead to hyper-methylation and to aberrant gene expression patterns [37]. Our data show that while loss of PRC2 leads to an increase in promoter DNA methylation at target genes, the modest increase is not sufficient to effect large changes in gene expression. The low level of DNA methylation observed at the genes that show decreased H327me3 may interfere with the proper activation developmental pathways or may lead to epigenetic instability in differentiated cell types. Consistent with the latter idea, a low level of seeding of DNA methylation can lead to an accumulation of this modification over time [37] .

In addition to its roles in development, faulty regulation of PcG proteins has been strongly correlated with the progression and severity of cancer. In many different types of cancer, PcG proteins, such as EZH2, are expressed at higher than normal levels, which is thought to lead to aberrant silencing of tumor suppressor genes [40]–[43]. Indeed, forced overexpression of *Ezh2* leads to cancer phenotypes [44], and inhibition of EZH2 is a promising cancer therapy [45], [46]. Conversely, decreased expression of PcG proteins has also been observed in tumor samples, such as the downregulation of *Bmi1* in melanoma [47], suggesting that loss of Polycomb complexes leads to activation of oncogenes. Emerging evidence also indicates that perturbation of PcG proteins in cancer may have consequences on DNA methylation patterns. For example, PRC2 target genes in ESCs are more likely to show promoter DNA hypermethylation in cancer cells, suggesting that H2K27me3 marks genes that become targets for more permanent silencing [48]–[51]. These studies suggest that loss of PRC2 activity can ultimately lead to epigenetic instability and loss of cell identity during tumorigenesis. Thus, additional studies to investigate the diverse mechanisms that PcG proteins employ to regulate cell fate transitions and cell identity are critical to further our understanding of both normal and pathologic development, and to facilitate the design of relevant therapies.

## Conclusions

**Figure 6:**
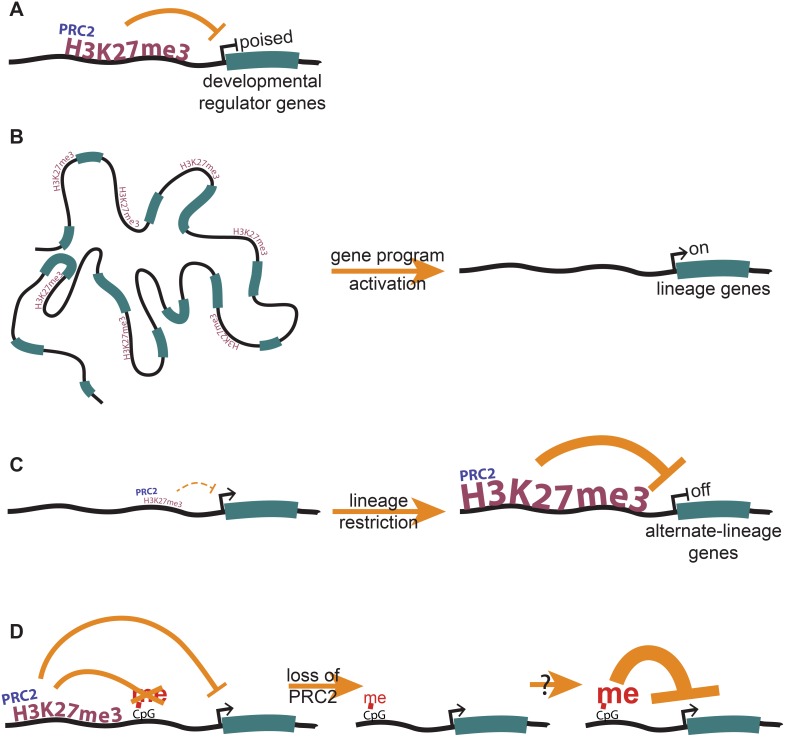
PRC2 plays roles in gene regulation both in pluripotency and during lineage commitment. (**A**) In ESCs, PRC2 localizes largely to developmental regulator genes, and maintains them in their repressed, and yet poised, state. (**B**) Proper H3K27me3 levels are necessary to activate developmental gene programs during differentiation. (**C**) A gain in H3K27me3 during differentiation represses alternate-lineage genes, allowing for efficient lineage restriction. (**D**) PRC2 antagonizes DNA methylation in *cis*, and may play a role in preventing the premature permanent repression of developmental genes.

Loss of H3K27me3 at gene promoters in ESCs leads to gene derepression in ESCs (Figure 6A), and an inability to properly activate developmental gene programs when signaled to do so (Figure 6B). We find that an inability to gain H3K27me3 over differentiation leads to failure to properly repress non-lineage programs, leading to defects in lineage restriction and cell fate (Figure 6C). We also show that PRC2/H3K27me3 is directly antagonistic to DNA methylation in *cis*. While loss of PRC2 does not lead to robust DNA methylation and repression of target genes (Figure 6D), we propose that the low level seeding of inappropriate DNA methylation may lead to further epigenetic instability in differentiated cells, which may explain the molecular underpinnings of PRC2 disruption in cancer. Our work provides novel insights into the role of PRC2 in mammalian development, and its effect on gene expression during lineage commitment.

## Materials and Methods

### ESC culture

ESCs were cultured on irradiated MEFs under standard ESC conditions. This includes E14 and *Suz12^GT^* (ola/129 background), obtained from the Helin lab [22]; *Suz12^Δ^* (C57/BL6 background), obtained from the Koseki lab [10]; and *Eed^null^* (BALB/cR1 background) [24], obtained from the Magnuson lab. ESCs were collected by trypsinization, incubation on cell a culture plate for 20 minutes to remove MEFs, and collection of the ESCs in suspension.

### Spinal Motor Neuron differentiation

SMN differentiation was performed as described [52]. Cells were collected at day 5, before the terminal differentiation stage, and trypsinized to single-cell suspension for use in other assays.

### RNA-seq

RNA was isolated using Trizol according to manufacturer’s instructions, including optional step in protocol. RNA quality was determined by Agilent Bioanalyzer. RNA-seq libraries were prepared as in [53]. A final round of size selection by Agencourt AMPure XP beads was performed to remove small fragments such as primers. Sequencing was run on either an Illumina GA-2 or Hi-Seq. For analysis, Bowtie v. 0.12.7, Tophat v1.3.2 and Cufflinks v 1.2.1 and Cuffdiff were utilized to determine the expression levels of genes [54], using a NCBIN37, ENSEMBL-based annotation and flags -p 4, -r 170, –segment-length 20 –segment-mismatches 1–solexa1.3-quals –no-novel-juncs. Cufflinks was guided using the same annotation as Tophat with flags -b -u -p 6.

### Contour plots and regression

Two-dimensional kernel density estimates (Figures 3B–C) were computed from bivariate data (typically E14 wt versus a mutant cell line) using the kde2d function from MASS library in the R statistical environment (v.3.02), with 50 grid points in each direction, and 14 levels were visualized. For regression analysis, gene expression or read coverage fold-changes were log2-transformed, and dependent variables were regressed using a generalized linear modelling framework (glm, with an identity link function) in the R statistical environment (v. 3.0.2). Segmented regression was performed on the resulting object using the “segmented” R package (v. 0.3–3.0), with a starting psi parameter (i.e. inflexion point) set at −0.5 for all analyses For comparison, locally-weighted regression (loess) was also performed using the stat package.

### ChIP-seq

Chromatin immunoprecipitation (ChIP) was performed based on the protocol as described in Lee et al., 2006 [55], with modifications and adaptations. Briefly, a Diagenode bioruptor was used for sonication of formaldehyde-crosslinked cells on high for 30 s on/30 s off for 45 cycles in sonication buffer (20 mM Tris-HCl; 150 mM NaCl; 2 mM EDTA; 0.1 mM SDS; 1% Triton X-100; protease inhibitor (Thermo-scientific)). In ChIPs to be sequenced, the Diagenode IP-star was also used for automation of the ChIP protocol, according to the manufacturer’s specifications. Antibodies used for ChIP are listed in Table S1 in File S1. After purification of DNA, samples were used for quantification via qPCR and/or used to prepare libraries for Illumina sequencing. Library preparation is performed essentially as described in Schmidt et al., 2009; the amplification and size selection steps are reversed in order, and size selection was performed using Agencourt Ampure XP beads [56]. Sequencing was run on an Illumina Hi-Seq (barcoded). Peak calling was performed as previously described [53]. All ChIPs described in this paper are listed in Table S3 in File S1.

### X-gal staining

Cells grown on cell culture plates were fixed for 4 minutes in fixing solution (4% formaldehyde, 0.5% glutaraldehyde, 0.1 M NaH_2_PO_4_, 0.1 M Na_2_HPO_4_), rinsed twice with PBS, and stained at 37°C until sufficiently colored, in staining solution (1 mg/mL X-gal, 5 mM K_3_Fe(CN)_6_, 5 mM K_4_Fe(CN)_6_•3H_2_O, 2 mM MgCl_2_, 1xPBS).

### Immunoprecipitations

Protein G Dynabeads (Life Technologies) were added to the appropriate antibody and incubated in PBS + BSA for 4 hours at 4°C. Concomitantly, cells were incubated in lysis buffer (50 mM hepes pH 7.2, 250 mM NaCl, 10% (vol/vol) glycerol, 2 mM EDTA, 0.1% (vol/vol) Nonidet P-40, protease inhibitor (Thermo-scientific)) [57] for 20 minutes on ice. In the middle of this lysis, the cells were briefly homogenized using a Tissue Tearor homogenizer. This lysate was then spun down 5 minutes at 16,000 g at 4°C to remove debris, and the supernatant used as input. 3% of the input was boiled 10 minutes in Laemmli buffer and set aside at −20°C. The bead mixture was then added to the input, and this rotated at 4°C for 4 hours. Beads were washed 3x with lysis buffer, resuspended in Laemmli buffer, and boiled for 10 minutes before removal of beads and analysis of supernatant by Western blot.

### shRNA-mediated knockdown of transcripts in ESCs

Oligonucleotides were designed such that when annealed, they would form dsDNA that would be transcribed into an RNA hairpin. Annealed hairpin dsDNA was ligated into the pLKO.1 vector. This construct was then co-transfected with packaging vectors into 293 cells, and the virus produced was filtered and used to infect ESCs. These infected cells were puromycin-selected before testing the knockdown level by qRT-PCR.

### Quantitative reverse transcriptase PCR

For expression analysis, RNA was extracted using Trizol according to manufacturer’s instructions, and cDNA was made using MMLV reverse transcriptase according to manufacturer’s instructions, with random hexamer primers. Quantitative reverse transcriptase PCR was performed on either cDNA or ChIP template using a Roche LightCycler 480 machine, using KAPA SYBR FAST Master Mix (2X) optimized for this machine. Primers are listed in Table S2 in File S1. Reactions were prepared in triplicate and temperature cycled according to the product specifications. Analysis of data was performed by comparing each reaction of the experimental triplicate to each reaction of the control triplicate, using a 2^−dCp^ model [58]. The average and standard deviation of this set of results was then calculated.

### Immunohistochemistry

Aggregated motor neurons at day 5 of the Spinal Motor Neuron differentiation were collected and fixed for 20 minutes in 10% formalin, washed with PBS, and then dehydrated in sequentially higher concentrations of ethanol for 20 minutes each (70%, 80%, 95%, 95%, 100%, 100%, 100%) and washed three times in Xylene. They were then embedded in paraffin overnight at 60°C and sectioned to 0.4 uM. Parafin was removed with xylene, and the samples were rehydrated. Immunohistochemistry was performed with anti-OLIG2 antibody (Table S2 in File S1) at 1∶500.

### RRBS

Reduced representation bisulfite sequencing was performed as published [32] and sequenced on an Illumina Hi-Seq. The sequencing data were analyzed initially as published. Briefly, reads were mapped against an *in-silico* modified mouse genome (UCSC mm9, with inferred MspI restriction and genome-wide conversion of C to T and G to A) using maq (v 0.7.1–9) with the parameters -D -s 0 -M c -e 100 C. Resulting bam files were sorted and indexed with samtools (v.0.1.16, r963∶234) and per-position read pileups were obtained with mpileup using unmodified mm9 as a reference. Following that, for each sample, per-base read coverage and fraction of C or G-containing reads (depending on the read mapping strand) were extracted and CpG sites were summed and summarized using custom perl scripts. Genome-wide methylation levels were assessed by tallying the fraction of methylation-representative reads over read coverage in each sample for sites with 10x or higher coverage. For pairwise sample comparisons, sites meeting a 10x-read coverage in both samples were binned according to their methylation levels in both samples and displayed in matrix form. To assess relative over- or underrepresentation of a given bin, expected counts per bin were estimated by averaging pairwise replicate methylation matrices in all cell types (background model). Deviations from the expected distribution are therefore represented as the observed: expected ratios (fold enrichment). All DNA methylation values were floored at 0.01% to allow calculations for CpGs with no methylation. Association of a CpG with a gene was determined by its proximity. Briefly, a CpG located within 4 kb of a gene body was associated with that gene. Proximity to another gene-associated CpG was also used as an alternate criterion. Otherwise, it was assigned to the nearest PRC2 target gene within 200 kb.

All relevant data sets have been deposited at the NCBI Gene Expression Omnibus, under accession number GSE53508.

## Supporting Information

Figure S1 **The** ***Suz12^GT^*** **allele produces a truncation-fusion protein that interacts with canonical PRC2 components to form a partially functional complex. (A)** RNA-seq data shows the expected *Suz12* mRNA in *Suz12^GT^*, *Suz12^Δ^*, and wild-type (wt) ESCs. **(B)** Cell lysates from wt, *Suz12^GT^*, *Suz12^Δ^*, and *Eed^null^* ESCs were subjected to SDS-PAGE and western blotting with an antibody recognizing the C-terminal region of SUZ12. β-actin is included as a loading control. **(C)** The entire immunoblot shown cropped in Figure 1C. **(D)** The immunoblot shown in Figure 1C/S1C was quantified using QuantityOne software. Amount of EZH2 detected was normalized to the amount in the wild-type 3% input sample. **(E)** The degraded EZH2 (marked as *) in the immunoblot shown in Figure 1C was quantified using QuantityOne software and plotted normalized to the highest amount. **(F)** qRT-PCR was used to measure the depletion of *Ezh2* (top panel), and *Ezh1* (bottom panel) with respect to *Suz12^GT^* ESCs expressing a scrambled control hairpin. Error bars represent the standard deviation of three technical replicates. **(G)** Three distinct H3K27me3 ChIP-seq experiments on *Suz12^GT^* ESCs show a similar localization pattern with respect to wt ESCs and *Eed^null^* ESCs, as shown here at representative PRC2 target gene *Bmp2*. **(H)** ChIP-seq signal is shown in density plots at the TSS +/−2 kb. Each horizontal line is one PRC2 target gene. Reads per million in 50 bp bins is represented on a white to black scale, with black being the 95^th^ percentile value. Genes were sorted with respect to wt H3K27me3 signal.
(TIF)

Figure S2 ***Suz12^GT^*** **cells maintain some H3K27me3 at PRC2 target genes upon differentiation.** **(A)** RNA-seq FPKM values for *Pou5f1* (*Oct4*) are plotted for wt, *Suz12^GT^*, *Suz12^Δ^*, and *Eed^null^* ESCs and day 5 SMNs. **(B)** Metagene analysis of H3K27me3 ChIP-seq data in day 5 SMNs. Only PRC2 target genes are included in the analysis. Alternate representation of the bottom panel of Figure 2D with a smaller-scale y-axis is included to permit visualization of the differences between the three PRC2 mutant cell lines. **(C)** For all genes that are bivalent (H3K27me3+/H3K4me3+) in either ESCs or differentiated cells for the relevant cell type, log_2_-transformed fold-changes of H3K27me3 and H3K4me3 levels in TSS regions between D0 (ESCs) and D5 (SMN-lineage differentiated), respectively, are depicted in WT (left) and *Suz12^GT^* (right) cells. Genes that displayed a fourfold or greater increase H3K27me3 levels in WT cells are highlighted in red in both panels, while genes with a 1.5-fold drop in H3K27me3 levels and 1.5 fold or greater increase in H3K4me3 levels in WT are highlighted in blue.
(TIF)

Figure S3 ***Suz12^GT^*** **ESCs show diminished capacity to repress alternate lineage genes during lineage commitment. (A)** RNA-seq was performed on wild-type (wt), *Suz12^GT^*, Suz12^Δ^, and *Eed^null^* ESCs. The distribution of the fpkms of all genes are plotted here; the median is indicated and labeled for each cell type. The box extends through the InterQuartile Region (IQR): the 25^th^ to 75^th^ percentile. The whiskers represent 1.5x the length of the IQR. **(B)** RNA-seq and H3K27me3 ChIP-seq are shown for *Suz12^GT^* (left panel), *Suz12^Δ^* (middle panel), and *Eed^null^* (right panel) ESCs with respect to wt. Kernel densities of the data are represented as contour plots along 14 levels. Three regression methods were used to calculate localized best-fit, and are included for comparison. Simple linear regression is in red, loess is in green, and segmented regression (as in Figure 3B) in blue. The y = x line is in yellow. **(C)** Transcriptome analysis of wt, *Suz12^GT^*, *Suz12^Δ^*, and *Eed^null^* ESCs and day 5 SMNs using RNA-Seq. y-axis shows log_2_ of the ratio of FPKM in differentiated: ESC in mutant lines as indicated; x-axis represents this ratio in wt cells. Contour plots and regressions were generated as in S3B. **(D)** RNA-seq was performed on wt, *Suz12^GT^*, *Suz12^Δ^*, and *Eed^null^* ESCs and day 5 MNs. Only wt ESC PRC2 target genes are shown here to visualize how the expression of this set of genes changes over differentiation in PRC2 mutant versus wt cells. The y-axis of each panel shows the log_2_ of the ratio of the FPKM in differentiated vs. ESC in the respective mutant line; the x-axis shows the same value in the wt line. The y = x line is also plotted in orange for visual reference. As a large number of genes are represented here, data points were rendered transparent such that the density of points plotted in one place can be approximated by the opacity of the signal. **(E)** Relationship between change in H3K27me3 and expression over differentiation in wild-type cells is shown as box plots. All genes were binned by change in H3K27me3 levels over differentiation (log_2_ of H3K27me3 (day 5/day 0)). y-axis shows distribution of change in expression (log_2_ of FPKM (day 5/day 0)). **(F)** Gene list from wt quintiles (in part E) (shown here in gray) was used to generate box plots with *Suz12^GT^* expression data (shown in violet). The two cell types were superimposed to demonstrate their differences and similarities. **(G)** Example genes that show changes in expression and H3K27me3 levels over SMN differentiation in wt and *Suz12^GT^* cells are depicted. *Sox3* and *Nes* are expressed in neurectoderm. *Sox17* and *Gata4* are expressed in endoderm. *T* and *Bmp4* are expressed in mesoderm.
(TIF)

Figure S4 **DNA methylation is gained at some PRC2 target sites in PRC2 null mutant ESCs.** Data for *Suz12* ^Δ^ and *Eed^null^* ESCs are shown here (goes with Figure 4C). CpGs from regions enriched for H3K27me3 in wild-type (wt) ESCs are used. CpGs are binned according to their % methylation in wt ESCs on the y-axis and % DNA methylation in mutant ESCs on the x-axis.
(TIF)

File S1 **Excel workbook including four Supporting Tables (Table S1–S4).** Table S1. Antibodies. Table S2. Oligos. Table S3. ChIPs. Table S4. Gene Ontology.
(XLSX)
