# Supplementary figures and images for: Polycomb Repressive Complex 2 Regulates Lineage Fidelity during Embryonic Stem Cell Differentiation
Source: PLoS One. 2014 Oct 21;9(10):e110498. doi: 10.1371/journal.pone.0110498 (PMC4204901; doi:10.1371/journal.pone.0110498)

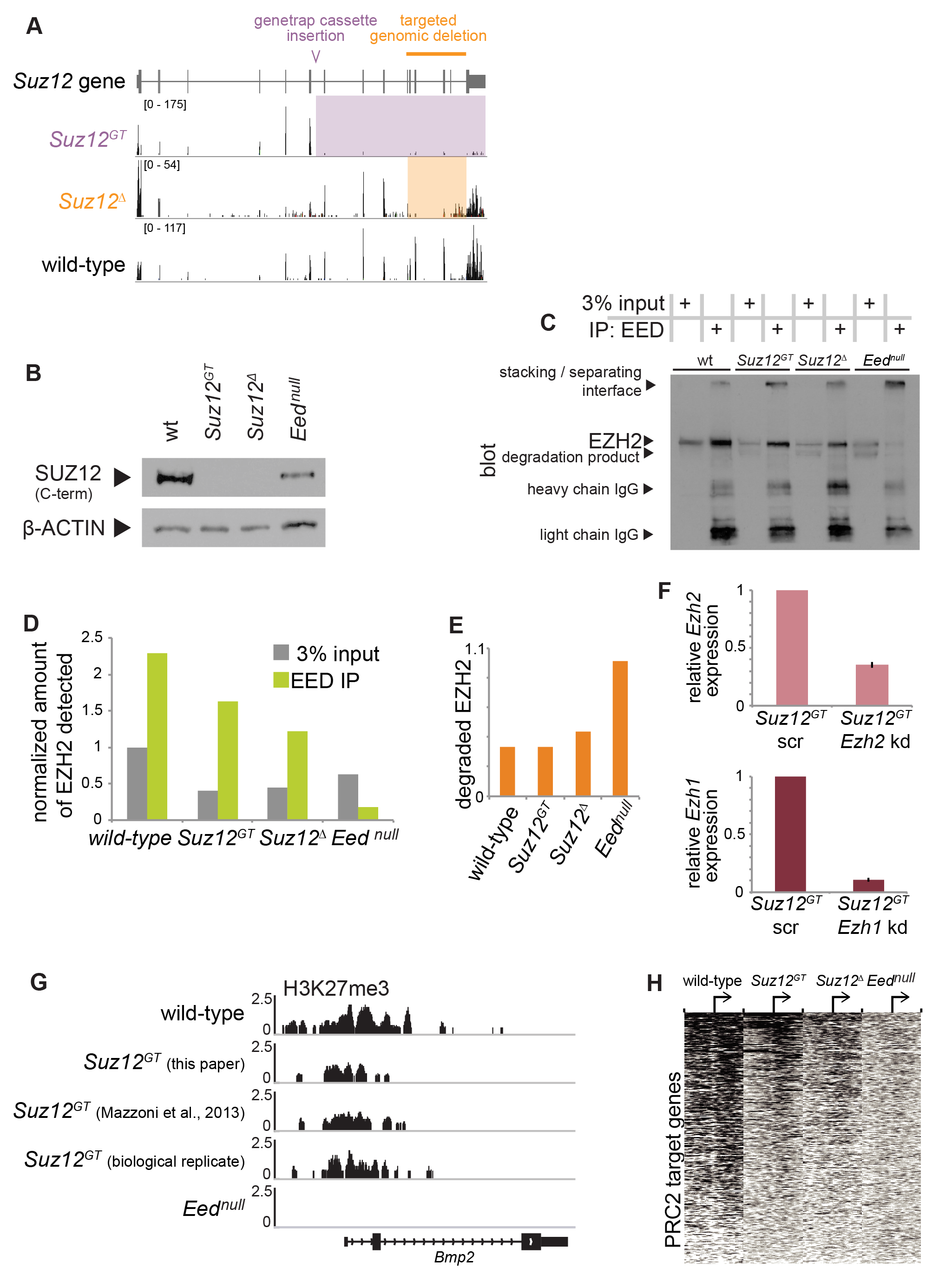

Supplement: Figure S1 — The Suz12GT allele produces a truncation-fusion protein that interacts with canonical PRC2 components to form a partially functional complex. (A) RNA-seq data shows the expected Suz12 mRNA in Suz12GT, Suz12Δ, and wild-type (wt) ESCs. (B) Cell lysates from wt, Suz12GT, Suz12Δ, and Eednull ESCs were subjected to SDS-PAGE and western blotting with an antibody recognizing the C-terminal region of SUZ12. β-actin is included as a loading control. (C) The entire immunoblot shown cropped in Figure 1C. (D) The immunoblot shown in Figure 1C/S1C was quantified using QuantityOne software. Amount of EZH2 detected was normalized to the amount in the wild-type 3% input sample. (E) The degraded EZH2 (marked as *) in the immunoblot shown in Figure 1C was quantified using QuantityOne software and plotted normalized to the highest amount. (F) qRT-PCR was used to measure the depletion of Ezh2 (top panel), and Ezh1 (bottom panel) with respect to Suz12GT ESCs expressing a scrambled control hairpin. Error bars represent the standard deviation of three technical replicates. (G) Three distinct H3K27me3 ChIP-seq experiments on Suz12GT ESCs show a similar localization pattern with respect to wt ESCs and Eednull ESCs, as shown here at representative PRC2 target gene Bmp2. (H) ChIP-seq signal is shown in density plots at the TSS +/−2 kb. Each horizontal line is one PRC2 target gene. Reads per million in 50 bp bins is represented on a white to black scale, with black being the 95th percentile value. Genes were sorted with respect to wt H3K27me3 signal. (TIF) [file pone.0110498.s001.tif]

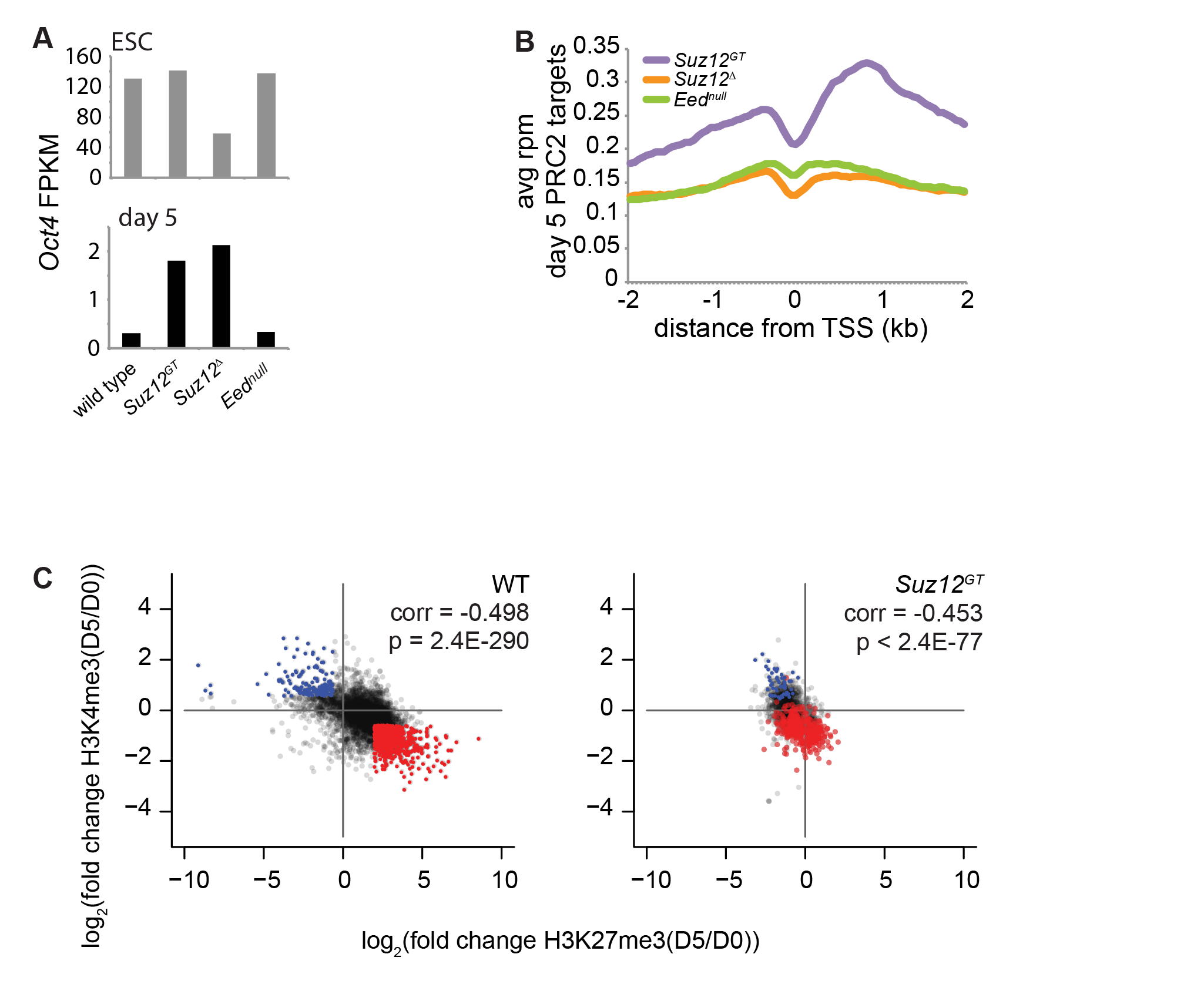

Supplement: Figure S2 — Suz12GT cells maintain some H3K27me3 at PRC2 target genes upon differentiation. (A) RNA-seq FPKM values for Pou5f1 (Oct4) are plotted for wt, Suz12GT, Suz12Δ, and Eednull ESCs and day 5 SMNs. (B) Metagene analysis of H3K27me3 ChIP-seq data in day 5 SMNs. Only PRC2 target genes are included in the analysis. Alternate representation of the bottom panel of Figure 2D with a smaller-scale y-axis is included to permit visualization of the differences between the three PRC2 mutant cell lines. (C) For all genes that are bivalent (H3K27me3+/H3K4me3+) in either ESCs or differentiated cells for the relevant cell type, log2-transformed fold-changes of H3K27me3 and H3K4me3 levels in TSS regions between D0 (ESCs) and D5 (SMN-lineage differentiated), respectively, are depicted in WT (left) and Suz12GT (right) cells. Genes that displayed a fourfold or greater increase H3K27me3 levels in WT cells are highlighted in red in both panels, while genes with a 1.5-fold drop in H3K27me3 levels and 1.5 fold or greater increase in H3K4me3 levels in WT are highlighted in blue. (TIF) [file pone.0110498.s002.tif]

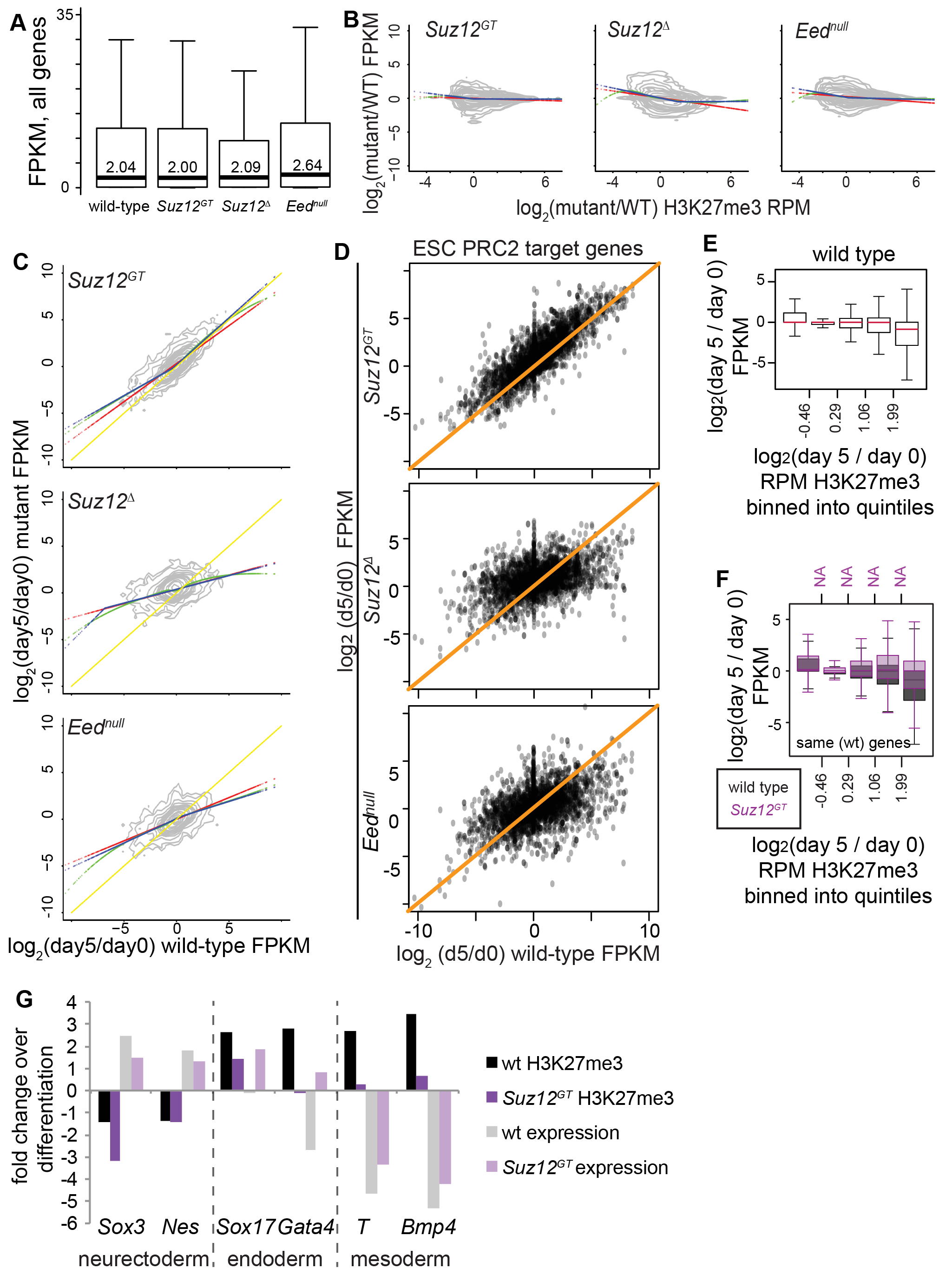

Supplement: Figure S3 — Suz12GT ESCs show diminished capacity to repress alternate lineage genes during lineage commitment. (A) RNA-seq was performed on wild-type (wt), Suz12GT, Suz12Δ, and Eednull ESCs. The distribution of the fpkms of all genes are plotted here; the median is indicated and labeled for each cell type. The box extends through the InterQuartile Region (IQR): the 25th to 75th percentile. The whiskers represent 1.5x the length of the IQR. (B) RNA-seq and H3K27me3 ChIP-seq are shown for Suz12GT (left panel), Suz12Δ (middle panel), and Eednull (right panel) ESCs with respect to wt. Kernel densities of the data are represented as contour plots along 14 levels. Three regression methods were used to calculate localized best-fit, and are included for comparison. Simple linear regression is in red, loess is in green, and segmented regression (as in Figure 3B) in blue. The y = x line is in yellow. (C) Transcriptome analysis of wt, Suz12GT, Suz12Δ, and Eednull ESCs and day 5 SMNs using RNA-Seq. y-axis shows log2 of the ratio of FPKM in differentiated: ESC in mutant lines as indicated; x-axis represents this ratio in wt cells. Contour plots and regressions were generated as in S3B. (D) RNA-seq was performed on wt, Suz12GT, Suz12Δ, and Eednull ESCs and day 5 MNs. Only wt ESC PRC2 target genes are shown here to visualize how the expression of this set of genes changes over differentiation in PRC2 mutant versus wt cells. The y-axis of each panel shows the log2 of the ratio of the FPKM in differentiated vs. ESC in the respective mutant line; the x-axis shows the same value in the wt line. The y = x line is also plotted in orange for visual reference. As a large number of genes are represented here, data points were rendered transparent such that the density of points plotted in one place can be approximated by the opacity of the signal. (E) Relationship between change in H3K27me3 and expression over differentiation in wild-type cells is shown as box plots. All genes were binned by change i [file pone.0110498.s003.tif]

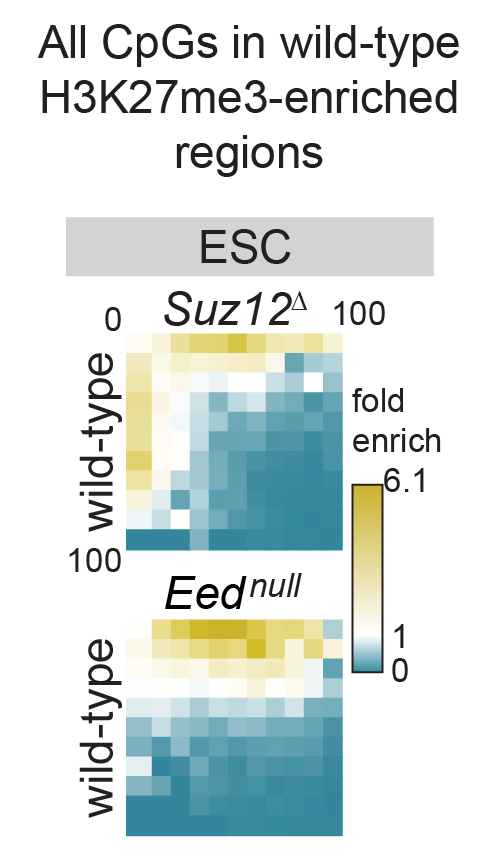

Supplement: Figure S4 — DNA methylation is gained at some PRC2 target sites in PRC2 null mutant ESCs. Data for Suz12 Δ and Eednull ESCs are shown here (goes with Figure 4C). CpGs from regions enriched for H3K27me3 in wild-type (wt) ESCs are used. CpGs are binned according to their % methylation in wt ESCs on the y-axis and % DNA methylation in mutant ESCs on the x-axis. (TIF) [file pone.0110498.s004.tif]
